# Effectiveness and Safety of mRNA Vaccines in the Therapy of Glioblastoma

**DOI:** 10.3390/jpm14090993

**Published:** 2024-09-19

**Authors:** Zdeslav Strika, Karlo Petković, Robert Likić

**Affiliations:** 1 School of Medicine, University of Zagreb, 10000 Zagreb, Croatia; zdeslav.strika@student.mef.hr (Z.S.); kpetkovic@student.mef.hr (K.P.); 2 Division of Clinical Pharmacology and Therapeutics, Department of Internal Medicine, Clinical Hospital Centre Zagreb, 10000 Zagreb, Croatia

**Keywords:** cancer immunotherapy, glioblastoma, lipid nanoparticles, mRNA vaccines, tumor microenvironment

## Abstract

Glioblastoma (GBM) is the most common and most malignant primary brain tumor, presenting significant treatment challenges due to its heterogeneity, invasiveness, and resistance to conventional therapies. Despite aggressive treatment protocols, the prognosis remains poor, with a median survival time of approximately 15 months. Recent advancements in mRNA vaccine technology, particularly the development of lipid nanoparticles (LNPs), have revitalized interest in mRNA-based therapies. These vaccines offer unique advantages, including rapid production, personalization based on tumor-specific mutations, and a strong induction of both humoral and cellular immune responses. mRNA vaccines have demonstrated potential in preclinical models, showing significant tumor regression and improved survival rates. Early-phase clinical trials have indicated that mRNA vaccines are safe and can induce robust immune responses in GBM patients. Combining mRNA vaccines with other immunotherapeutic approaches, such as checkpoint inhibitors, has shown synergistic effects, further enhancing their efficacy. However, challenges such as optimizing delivery systems and overcoming the immunosuppressive tumor microenvironment remain. Future research should focus on addressing these challenges and exploring combination therapies to maximize therapeutic benefits. Large-scale, randomized clinical trials are essential to validate the efficacy and safety of mRNA vaccines in GBM therapy. The potential to reshape the tumor microenvironment and establish long-term immunological memory underscores the transformative potential of mRNA vaccines in cancer immunotherapy.

## 1. Introduction

Glioblastoma (GBM), the most common and most malignant primary brain tumor, poses significant treatment challenges due to its heterogeneity, invasiveness, and resistance to conventional therapies [1]. The prognosis for GBM patients remains dismal, with a median survival time of approximately 15 months despite aggressive treatment protocols, including surgery, radiation, and chemotherapy [1]. Current treatment options include maximal safe resection followed by concurrent radiotherapy and temozolomide chemotherapy, but these approaches are limited by issues such as incomplete tumor resection, radiation resistance, and significant side effects of chemotherapy. Therefore, innovative treatment strategies are paramount.

## 2. Current Challenges in GBM Therapy

The development of therapies for GBM is hampered by several key challenges. Firstly, the tumor’s highly infiltrative nature makes complete surgical resection very difficult, leading to inevitable recurrence. Secondly, GBM exhibits profound resistance to both radiation and chemotherapy, which are the standard adjuvant therapies. This resistance is partly due to the tumor’s ability to repair DNA damage and its highly immunosuppressive microenvironment, which hinders the efficacy of immune-based therapies. Furthermore, the blood–brain barrier (BBB) restricts the delivery of therapeutic agents to the tumor site, significantly limiting the effectiveness of systemic therapies.

In addition to these challenges, recent studies have highlighted the role of metabolic reprogramming in GBM, which contributes to the aggressive nature of the tumor. One key finding is the identification of Praja2, a RING (Really Interesting New Gene) E3 ubiquitin ligase, as a critical regulator of GBM cell growth and metabolism. Praja2 promotes the degradation of kinase suppressor of Ras 2 (KSR2), leading to the downregulation of AMP-dependent protein kinase (AMPK) activity. This results in a metabolic switch from oxidative phosphorylation to glycolysis, supporting rapid tumor growth. Targeting Praja2 has shown therapeutic potential in preclinical models, where the use of transferrin-targeted self-assembling nanoparticles (SANPs) loaded with siRNA against Praja2 effectively inhibited GBM growth and improved survival in mouse models [2].

### 2.1. Tumor Microenvironment and Immune System Interaction in GBM

The tumor microenvironment (TME) of GBM is characterized by its highly immunosuppressive nature, which plays a critical role in tumor progression and resistance to therapy. The GBM TME consists of a complex network of immune cells, including tumor-associated macrophages (TAMs), regulatory T cells (Tregs), and myeloid-derived suppressor cells (MDSCs), all of which contribute to immune evasion and tumor growth. These cells secrete cytokines and other factors that suppress the activity of cytotoxic T lymphocytes (CTLs) and natural killer (NK) cells, thereby enabling the tumor to escape immune surveillance. Moreover, the presence of immunosuppressive molecules, such as programmed death-ligand 1 (PD-L1), further inhibits the anti-tumor immune response. Understanding the interplay between the GBM TME and the immune system is crucial for developing effective immunotherapeutic strategies, such as mRNA vaccines, which aim to modulate the immune response to target and eliminate tumor cells.

The immunosuppressive nature of the GBM tumor microenvironment (TME) presents significant challenges for therapeutic interventions, especially for immunotherapies like mRNA vaccines. A crucial component of this immunosuppression is the role of metabolic reprogramming in both tumor cells and the surrounding immune cells. GBM cells exhibit a metabolic switch to aerobic glycolysis (the Warburg effect), which not only supports their rapid growth but also alters the TME by promoting an acidic environment. This acidic microenvironment further suppresses the function of immune cells, particularly cytotoxic T lymphocytes (CTLs) and natural killer (NK) cells, whose activity is pH-sensitive. Additionally, tumor-associated macrophages (TAMs), which dominate the immune cell population in the GBM TME, often exhibit an M2-like phenotype. This phenotype is known for supporting tumor growth and inhibiting anti-tumor immune responses, as opposed to the M1 phenotype, which promotes inflammatory responses and tumor suppression. Addressing these metabolic and immunological aspects of the TME could enhance the efficacy of mRNA vaccines by reprogramming the immune cells to support anti-tumor activity.

### 2.2. History and Development of mRNA Vaccines in Oncology

The concept of using mRNA in vaccines dates back to the early 1990s, when mRNA was first considered as a potential tool for immunotherapy [3]. Early research demonstrated that mRNA could be used to express antigens in cells, thereby stimulating an immune response. However, initial efforts were hampered by technical challenges, including the instability of mRNA and difficulties in delivering it effectively to target cells. Over the subsequent decades, significant advancements in mRNA synthesis, delivery systems, and stabilization methods have revitalized interest in mRNA-based therapies.

One of the major breakthroughs came with the development of lipid nanoparticles (LNPs) as a delivery mechanism, which protects the mRNA from degradation and facilitates its uptake by cells [4]. This technology was crucial in the rapid development and deployment of mRNA vaccines against COVID-19, proving that mRNA vaccines could be safe, effective, and rapidly produced at scale. These advancements have opened new avenues for the application of mRNA technology in oncology.

### 2.3. Unique Advantages of mRNA Vaccines

mRNA vaccines offer several unique advantages over traditional therapies and other types of cancer vaccines. Table 1 compares the mechanisms of action of different types of vaccines, including mRNA vaccines. Firstly, they can be designed and produced quickly, allowing for a swift response to emerging diseases and the personalization of cancer treatments based on specific mutations in an individual’s tumor [5]. This rapid development cycle contrasts sharply with the more extended production timelines of traditional vaccines, such as protein or whole-cell vaccines, which often require months to years of development and manufacturing.

Secondly, unlike DNA-based vaccines, mRNA vaccines do not integrate into the host genome, reducing the risk of insertional mutagenesis and they degrade naturally after protein translation, minimizing long-term side effects [6]. In contrast, DNA vaccines carry a theoretical risk of genomic integration, which can potentially lead to mutagenesis or oncogenesis, albeit this risk remains largely hypothetical.

Thirdly, mRNA vaccines are highly effective at inducing both humoral and cellular immune responses, stimulating the production of specific antibodies and activating cytotoxic T lymphocytes (CTLs) that target and destroy cancer cells [7]. Traditional protein-based vaccines often predominantly elicit humoral responses, which, while crucial, may not be sufficient for combating cancers that require robust cellular immunity.

Additionally, mRNA vaccines can be tailored to express virtually any protein antigen, making them highly adaptable to different types of cancer and individual patient needs. This flexibility allows for the design of personalized cancer vaccines targeting patient-specific neoantigens [8]. Other cancer vaccines, such as peptide vaccines, may face limitations in antigenic diversity and the ability to evoke comprehensive immune responses. 

Lastly, as mRNA vaccines do not require the use of live pathogens, they are considered non-infectious and pose no risk of causing disease [9]. This safety profile stands in contrast to live-attenuated vaccines, which, while effective, carry a small risk of reversion to virulence, especially in immunocompromised individuals. 

In summary, the unique advantages of mRNA vaccines in oncology—rapid production, safety, robust induction of both humoral and cellular responses, adaptability for personalization, and ease of manufacturing—highlight their transformative potential compared to other cancer vaccine modalities.

## 3. Application of mRNA Vaccines in Glioblastoma

### 3.1. Mechanism of Action

**Figure 1:**
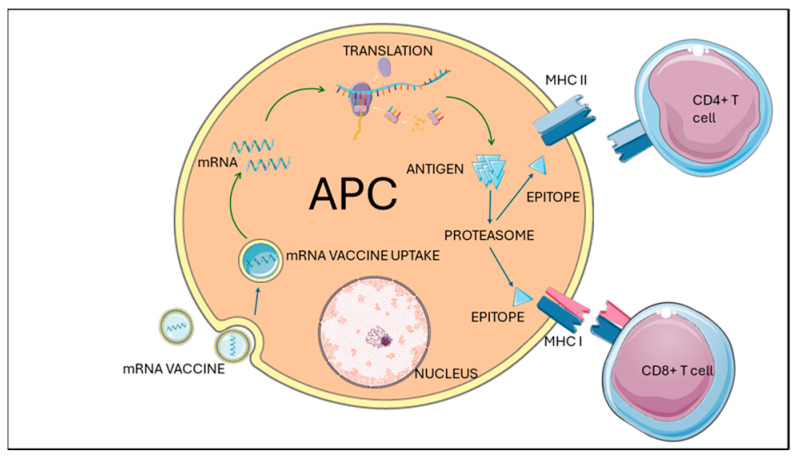
Mechanism of mRNA vaccine action in antigen-presenting cells (APCs) for glioblastoma treatment. This figure illustrates the intracellular processing and presentation of antigens from mRNA vaccines by antigen-presenting cells (APCs) and the subsequent activation of T cells. The mRNA vaccine enters the APC through endocytosis, depicted by the mRNA vaccine enclosed in lipid nanoparticles being taken up by the APC. Once inside the cytoplasm, the mRNA is released and translated by ribosomes into the encoded antigen protein. The synthesized antigen is then processed by the proteasome, which degrades the protein into smaller peptide fragments known as epitopes. Some of these epitopes are loaded onto major histocompatibility complex (MHC) class I molecules and transported to the cell surface, where they can be recognized by CD8+ T cells. This pathway is crucial for the activation of cytotoxic T lymphocytes (CTLs), which can directly kill glioblastoma cells. Other epitopes are loaded onto MHC class II molecules and transported to the cell surface to be recognized by CD4+ T helper cells. This interaction is essential for the activation and proliferation of helper T cells, which aid in orchestrating the immune response by stimulating other immune cells, including B cells and CTLs. The interaction between the MHC–epitope complex and the T cell receptor on CD4+ and CD8+ T cells leads to their activation. Activated CD8+ T cells can target and destroy glioblastoma cells presenting the specific antigen, while CD4+ T cells provide critical support to sustain and enhance the immune response. This comprehensive pathway highlights the critical steps involved in the immune response elicited by mRNA vaccines and their potential application in glioblastoma treatment by harnessing the body’s own immune system to target and eliminate cancer cells. Figure 1 was drawn in part using images from Servier Medical Art. Servier Medical Art by Servier is licensed under a Creative Commons Attribution 4.0 License.

The principle behind mRNA vaccines in oncology involves encoding tumor-associated antigens (TAAs) or neoantigens in mRNA sequences, which, when introduced into the body, are translated into proteins by host cells. These proteins are then presented on the cell surface, triggering an immune response aimed at targeting and destroying cancer cells [3,4]. This approach leverages the body’s own machinery to produce antigens, offering a personalized and highly adaptable method of cancer treatment, see Figure 1.

The design of mRNA vaccines involves several critical components. The mRNA sequence is synthesized to include coding regions for the target antigens, untranslated regions (UTRs) that enhance stability and translation, and a poly(A) tail that protects against degradation [3]. The effective delivery of mRNA into cells is crucial, and lipid nanoparticles (LNPs) are commonly used to encapsulate mRNA, facilitating its entry into cells and protecting it from degradation [10]. Immunostimulatory elements such as adjuvants may be included to boost the immune response by mimicking pathogenic signatures and enhancing the vaccine’s efficacy [11].

One of the significant challenges in delivering mRNA vaccines to brain tumors like glioblastoma is crossing the blood–brain barrier (BBB). The BBB is a highly selective barrier that prevents most molecules from entering the brain, posing a substantial obstacle for the delivery of therapeutic agents. Several strategies are being developed to address this issue, see Table 2.

Firstly, the use of focused ultrasound (FUS) in combination with microbubbles has shown promise in transiently disrupting the BBB, allowing the passage of therapeutic agents, including mRNA encapsulated in nanoparticles [12]. This technique uses targeted ultrasound waves to temporarily open the tight junctions of the BBB, facilitating drug delivery to the brain.

Secondly, receptor-mediated transcytosis is another innovative approach being explored. This method exploits endogenous transport mechanisms by attaching ligands or antibodies to nanoparticles that bind to specific receptors on the BBB, such as the transferrin or insulin receptors, facilitating the transport of encapsulated mRNA across the barrier [13].

Thirdly, the development of novel nanoparticle formulations that can naturally cross the BBB is under investigation. For example, nanoparticles can be designed with surface modifications that enhance their ability to traverse the BBB. PEGylation, the attachment of polyethylene glycol (PEG) chains to nanoparticles, is one such modification that increases the stability and circulation time of nanoparticles, enhancing their chances of crossing the BBB [14].

Another approach involves the use of cell-penetrating peptides (CPPs), which are short peptides that can facilitate the delivery of therapeutic agents across cellular membranes, including the BBB. CPPs can be conjugated to nanoparticles carrying mRNA, improving their delivery efficiency to brain tissues [15].

Additionally, ongoing research is focused on utilizing viral vectors that have a natural ability to cross the BBB. Adeno-associated viruses (AAVs) and lentiviruses are being engineered to deliver mRNA directly to brain cells, taking advantage of their efficient transduction capabilities [16].

Despite these advancements, ensuring the stability and controlled release of mRNA once it reaches the target site remains a challenge. Novel hydrogel-based delivery systems are being developed to provide sustained release of mRNA, enhancing its therapeutic efficacy while minimizing potential side effects [17].

### 3.2. Immune Responses

The immune response elicited by mRNA vaccines involves multiple components of the immune system, playing a crucial role in their efficacy against tumors like glioblastoma. When mRNA vaccines are administered, they induce the production of specific antibodies against the target antigens. These antibodies can neutralize tumor cells and mark them for destruction by other immune cells, such as macrophages and natural killer (NK) cells [2]. 

The primary mechanism of action for mRNA vaccines in oncology is through the activation of cytotoxic T lymphocytes (CTLs), which recognize and kill tumor cells presenting the antigen on MHC class I molecules. This cellular response is critical for targeting and eliminating cancer cells [5,6].

Recent studies have highlighted the potential of mRNA vaccines to reshape the tumor microenvironment (TME). They can enhance the infiltration of immune cells into the tumor, counteract immunosuppressive factors within the TME, and promote a pro-inflammatory milieu that supports anti-tumor immunity [4,8]. This ability to modulate the TME is critical for overcoming the immunosuppressive barriers that often limit the efficacy of traditional therapies. mRNA vaccines also establish immunological memory, providing long-term protection against cancer recurrence. Memory T cells generated during the initial response can quickly respond to subsequent encounters with the antigen [7].

Comparatively, traditional cancer therapies, such as chemotherapy and radiation, primarily aim to kill cancer cells directly but often do so at the expense of damaging healthy tissues and weakening the immune system. In contrast, mRNA vaccines leverage the body’s own immune system to target and destroy cancer cells, offering a more specific and potentially less harmful approach. 

### 3.3. Clinical Outcomes

Animal models of GBM have demonstrated significant tumor regression and improved survival rates following mRNA vaccine administration, providing proof-of-concept for their use in treating GBM and laying the groundwork for clinical trials [18]. Several preclinical and clinical studies have reported positive outcomes with mRNA vaccines in GBM therapy, as summarized in Table 3.

Early-phase clinical trials in humans have shown that mRNA vaccines are safe and capable of inducing robust immune responses in GBM patients. For example, a Phase I clinical trial conducted in 2013 involved personalized mRNA vaccines targeting patient-specific neoantigens in patients with newly diagnosed glioblastoma. This study enrolled 16 patients, and the vaccines were well-tolerated, with no serious adverse events reported. Notably, 8 out of 16 patients showed a prolonged progression-free survival (PFS) beyond the expected median for GBM, indicating a potential therapeutic benefit [19].

Another promising trial is the ongoing Phase Ib study by the University of Florida, which evaluated the efficacy of the personalized mRNA vaccine in 4 patients with advanced GBM. Early results have shown encouraging signs of tumor shrinkage and enhanced immune responses [20].

While early-phase clinical trials for mRNA vaccines in GBM have shown promising results in terms of safety and the induction of robust immune responses, several obstacles remain, preventing the achievement of consistent clinical efficacy. A significant factor is the variability in neoantigen expression between patients. GBM is highly heterogeneous, and the mutational burden can differ greatly even within different regions of the same tumor. This heterogeneity complicates the development of standardized vaccines and necessitates highly personalized approaches, which may not always be feasible on a large scale. Furthermore, the durability of the immune response generated by mRNA vaccines needs further investigation. While preclinical models show promise in inducing long-term immunological memory, clinical trials must demonstrate that this memory translates into prolonged progression-free survival and overall survival in patients.

Combining mRNA vaccines with other immunotherapeutic approaches, such as checkpoint inhibitors and dendritic cell vaccines, has shown synergistic effects, further enhancing their efficacy. These combination therapies aim to overcome the immunosuppressive nature of the TME and boost overall treatment outcomes [22]. For instance, a study combining mRNA vaccines with standard surgery and chemoradiotherapy demonstrated enhanced T cell activation and tumor regression in a phase I trial in GBM patients [21].

Despite these promising results, several challenges remain in the clinical translation of mRNA vaccines for GBM. These include optimizing delivery systems, overcoming the immunosuppressive tumor microenvironment, and ensuring vaccine stability and efficacy [23].

Future research should focus on addressing these challenges and exploring combination therapies to maximize therapeutic benefits [24]. Large-scale, randomized clinical trials with diverse patient populations are essential to validate the efficacy and safety of mRNA vaccines in GBM therapy. Additionally, advancements in delivery technologies and the identification of novel neoantigens could further enhance the clinical outcomes of these promising treatments.

## 4. The Way Forward

mRNA vaccines hold significant promise for the treatment of glioblastoma (GBM). The mechanisms of action, involving the delivery and expression of tumor-associated antigens (TAAs) or neoantigens, have been well elucidated, showing that these vaccines can elicit strong and specific immune responses. Studies have demonstrated the ability of mRNA vaccines to activate both humoral and cellular immunity, including the establishment of immunological memory, which is crucial for long-term cancer control [25]. Additionally, the potential to modulate the tumor microenvironment to favor an anti-tumor response represents a significant advancement [10].

While this commentary provides a comprehensive coverage of recent advances in mRNA vaccine technology and their application to glioblastoma, we synthesized findings from a variety of studies, providing a broad perspective on the current state of research. The focus on both preclinical and clinical outcomes offers a balanced view of the potential and challenges of this therapeutic approach [22].

However, our reliance on published studies means that unpublished data and ongoing trials were not taken into account, potentially overlooking emerging developments. Furthermore, the heterogeneity of the studies in terms of design, sample size, and endpoints makes it difficult to draw definitive conclusions about the efficacy of mRNA vaccines in GBM therapy [24]. Future research should aim to address these gaps by standardizing study protocols and including larger, more diverse patient populations to validate the findings [26].

In conclusion, mRNA vaccines represent a promising therapeutic approach for glioblastoma, capable of eliciting strong and specific immune responses against tumor cells. Significant progress has been made, yet further research is needed to overcome existing challenges, optimize delivery systems, and explore combination therapies. The potential to reshape the tumor microenvironment and establish long-term immunological memory highlights the transformative potential of mRNA vaccines in cancer immunotherapy [3].

## Figures and Tables

**Table 1:** Vaccine mechanisms.

Vaccine Type	Mechanism	Examples
Inactivated vaccines	Contain killed or inactivated viruses/bacteria	Hepatitis A, polio (IPV), influenza (some formulations)
Live-attenuated vaccines	Contain weakened live viruses/bacteria	Measles, mumps, rubella (MMR), varicella (chickenpox), yellow fever
Subunit, recombinant, polysaccharide, and conjugate vaccines	Use parts of the pathogen (proteins, sugars, etc.)	Human papillomavirus (HPV), hepatitis B, pneumococcal (conjugate), meningococcal (polysaccharide or conjugate)
Toxoid vaccines	Contain inactivated toxins (toxoids)	Diphtheria, tetanus
mRNA vaccines	Deliver mRNA to produce a pathogen protein; potential for rapid production, strong immune responses, and adaptability for personalization	COVID-19 vaccines (Pfizer-BioNTech, Moderna)
Viral vector vaccines	Use a modified virus to deliver pathogen genetic material	COVID-19 vaccines (AstraZeneca, Johnson & Johnson)
DNA vaccines (under development)	Inject DNA that encodes a pathogen antigen	Currently experimental, research ongoing
Protein subunit vaccines	Include harmless pieces of the pathogen	Novavax COVID-19 vaccine

**Table 2:** List of strategies utilized to address the effects of BBB in mRNA vaccine delivery to brain tumors.

Strategy	Description	Chronological Development	References
Focused ultrasound (FUS)	Uses targeted ultrasound waves in combination with microbubbles to transiently disrupt the BBB, allowing the passage of therapeutic agents.	Developed in the early 2000s	Anastasiadis et al., 2021 [12]
Receptor-mediated transcytosis	Exploits endogenous transport mechanisms by attaching ligands or antibodies to nanoparticles that bind to specific receptors on the BBB, facilitating transport.	Emerged in the late 2000s	Fan et al., 2014 [13]
Nanoparticle modifications	Involves designing nanoparticles with surface modifications, such as PEGylation, to enhance their ability to cross the BBB and increase stability.	Progressed during the 2010s	Alexander et al., 2019 [14]
Cell-penetrating peptides (CPPs)	Short peptides that facilitate the delivery of therapeutic agents across cellular membranes, including the BBB, improving delivery efficiency.	Gained traction in the 2010s	Suk et al., 2016 [15]
Viral vectors	Utilizes viral vectors such as Adeno-associated viruses (AAVs) and lentiviruses that naturally cross the BBB to deliver mRNA directly to brain cells.	Became prominent in the 2020s	Bulcha et al., 2021 [16]
Hydrogel-based systems	Novel systems that provide sustained release of mRNA, enhancing therapeutic efficacy while minimizing side effects once mRNA reaches the target site.	Emerging in the early 2020s	Zhong et al., 2023 [17]

**Table 3:** Summary of clinical outcomes of mRNA vaccines in GBM therapy.

Study	Type of Study	Patient Population	Outcomes
Sayour et al. (2015) [18]	Preclinical	Animal models of GBM	Significant tumor regression, improved survival rates
Keskin et al. (2019) [19]	Phase I clinical trial	Newly diagnosed GBM patients	Prolonged progression-free survival in 8/16 patients, well-tolerated
University of Florida (ongoing) [20]	Phase Ib clinical trial	Advanced GBM patients	Early results show tumor shrinkage, enhanced immune responses
Ghiaseddin et al. (2023) [21]	Phase I/II clinical trial	GBM patients	Enhanced T cell activation, tumor regression

## Data Availability

Data is available from the corresponding author upon a reasonable request.
